# Paediatric Inflammatory Bowel Disease and its Relationship with the Microbiome

**DOI:** 10.1007/s00248-021-01697-9

**Published:** 2021-03-05

**Authors:** Rachel S. Fitzgerald, Ian R. Sanderson, Marcus J. Claesson

**Affiliations:** 1 School of Microbiology, University College Cork, Cork, Ireland; 2 APC Microbiome Ireland, University College Cork, Cork, Ireland; 3 Centre for Immunobiology, Blizard Institute, Barts and the London School of Medicine and Dentistry, Queen Mary University of London, London, UK

**Keywords:** Gut microbiota, Microbiome, Paediatric inflammatory bowel disease, Ulcerative colitis, Crohn’s disease, Review

## Abstract

Paediatric inflammatory bowel disease (IBD) is a chronic inflammatory disorder of the digestive tract, comprising of Crohn’s disease (CD), ulcerative colitis (UC), and, where classification is undetermined, inflammatory bowel disease unclassified (IBDU). Paediatric IBD incidence is increasing globally, with prevalence highest in the developed world. Though no specific causative agent has been identified for paediatric IBD, it is believed that a number of factors may contribute to the development of the disease, including genetics and the environment. Another potential component in the development of IBD is the microbiota in the digestive tract, particularly the gut. While the exact role that the microbiome plays in IBD is unclear, many studies acknowledge the complex relationship between the gut bacteria and pathogenesis of IBD. In this review, we look at the increasing number of studies investigating the role the microbiome and other biomes play in paediatric patients with IBD, particularly changes associated with IBD, varying disease states, and therapeutics. The paediatric IBD microbiome is significantly different to that of healthy children, with decreased diversity and differences in bacterial composition (such as a decrease in Firmicutes). Changes in the microbiome relating to various treatments of IBD and disease severity have also been observed in multiple studies. Changes in diversity and composition may also extend to other biomes in paediatric IBD, such as the virome and the mycobiome. Research into biome differences in IBD paediatric patients may help progress our understanding of the aetiology of the disease.

## Introduction

Crohn’s disease (CD) and ulcerative colitis (UC) are idiopathic inflammatory bowel diseases (IBD). Wilhelm Fabry discovered Crohn’s disease in 1623, and its features were more definitively described by T.K. Dalziel in Scotland in 1913 [1] though it was later described by and named Burril B Crohn in 1932. The British physician Sir Samuel Wilks was the first to describe ulcerative colitis in 1859 [2]. Both CD and UC can occur in adults and children. There are a number of proposed risk factors in the development of IBD. One such potential risk factor or perhaps, as some have suggested, even a causative agent, is perturbed microbiota in the intestine. This review will look at the role of the microbiome in paediatric patients with IBD, outlining the findings thus far and the potential future of this particular area of research.

## Pathology of Crohn’s Disease and Ulcerative Colitis

### Primary Effects

Although both Crohn’s disease (CD) and ulcerative colitis (UC) are chronic inflammatory disorders of the gastrointestinal tract, they have unique features which distinguish them as distinct diseases. UC is a mucosal disease which originates in the rectum and may extend proximally. The ileum is generally not involved, and diffuse inflammation (a microscopic feature of UC) is confined to the mucosa and submucosa [3, 4]. CD shows patchy and segmental distribution and may involve any part of the gastrointestinal tract [3–5], though most commonly presents in the ileum. Granulomas and fissures are microscopic features often distinguishing CD from UC [3, 5].

### Secondary Effects

In addition to the gastrointestinal symptoms, IBD is also associated with a set of additional risks. Patients with IBD are at an increased risk of cancer, particularly colon cancer (UC and CD) and small bowel cancer (CD) (for paediatric patients development of such cancers may occur when they reach adulthood). The risk between these intestinal cancers and IBD is influenced by multiple factors, including the extent of the disease, the age of the patient at diagnosis, and the length of time since the patient was diagnosed [5], suggesting the possibility that paediatric patients may be at an even greater risk given the permanency of the diseases. A study of Danish and Finnish paediatric IBD patients found them to be 2.5 times as likely to develop cancer as the general population [6], and similar findings were reported in a study of Swedish paediatric patients by Olen et al. [7]. Patients with IBD may also suffer with extra intestinal manifestations linked to IBD such as arthritis, liver disease, and skin or ocular complications. These manifestations are common in children and adults, but in children, they may precede gastrointestinal symptoms [5].

Paediatric patients are also at risk of growth failure [5], which occurs in approximately 40% of CD and 10% of UC paediatric patients [8]. Approximately, a fifth of paediatric CD patients achieved a height significantly less than the expected target (>8 cm below target height) [9]. Consequently, steroids used to treat UC and CD in adults may not always be appropriate in paediatric patients due to the risk of growth retardation associated with these medications, especially in cases of children already suffering malnutrition due to IBD. Malnutrition is a more common occurrence in CD than UC; however, in recent years, an increased number of children diagnosed with IBD are either overweight or obese, following global trends of the general paediatric population [10]. Rates of obesity for children with UC were similar to the general population; however, lower rates were observed in children with CD.

Furthermore, psychosocial stress is often an additional side effect of IBD in children and adults. Absence from school, isolation from peers, extended hospital stays, and familial stress may all be contributing factors to this. Children with IBD were found to be at a higher risk of depressive disorders [11] and more likely to report a lower health related quality of life [12]. Danish and Finnish IBD patients who had childhood onset IBD have been found to be more likely to die from suicide then the general population, which may be reflective of this increased probability of depressive disorders [6].

## Incidence and Prevalence of IBD

Prevalence of CD and UC are highest in the developed world, particularly North America and Europe [13], and the incidence of IBD appears to be increasing, particularly in developing countries [14]. Approximately, 20 to 25% of patients present with IBD before the age of 20 [5, 8, 15], with approximately 4% of paediatric IBD presenting before the age of 5, and 10% before the age of 10 [8, 15]. For paediatric IBD, many studies suggest an increasing prevalence of paediatric IBD, even in the developed world [16, 17]. A study of paediatric IBD in Ontario found that prevalence per 100,000 of the population had increased from 42.1 in 1994 to 56.3 in 2005 and incidence rates per 100,000 increased from 9.5 in 1994 to 11.4 in 2005 [18].

## Potential Risk Factors for IBD

A major risk factor influencing the incidence of IBD is its genetic component, with >200 IBD associated loci [19]. Familial studies have demonstrated the genetic link of IBD [20], with up to 14% of IBD patients having a family member also suffering from the disease [21]. These genetic links can be seen in Jewish populations, where both CD and UC are found to more common in people of Jewish descent [2], and familial risk being increased for Jews vs non-Jews [22].

Increased risk is also related to geography with the prevalence and incidence of IBD higher in developed countries though this is most likely due to environmental risk factors such as industrialisation (leading to increases in pollution, such as air pollution [23]), sanitation (the hygiene hypothesis [24, 25]), and western diet and lifestyle.

A potential risk factor in IBD which has seen increased interest is the role of the microbiota in IBD, particularly the gut microbiota. Studies involving the composition and abundance of microorganisms in the gut (or ‘gut microbiome’) in IBD have revealed differences between healthy individuals and patients with IBD [26], both in colon [27] and stool samples [28], as well as between different disease states and varying with disease severity [29–31]. These differences can be seen in both children and adult IBD patients; however, for the purpose of this review, the focus will be on paediatric IBD.

## The Paediatric Microbiome

The microorganisms that colonise the human gut are often collectively referred to as the gut microbiome. When a child is born, the gut is initially colonised by environmental microbes, mainly from the mother’s microbiome, that may be the vaginal (or faecal) microbiome or the skin microbiome if born via Caesarean section [32]. The phyla Firmicutes, Proteobacteria, Bacteroidetes, and the phylum Actinobacteria (especially the genus *Bifidobacterium*) generally colonise the infant gut [33, 34]. The two most common phyla in the adult gut, Bacteroidetes and Firmicutes, generally dominate by the end of the first year of the infant’s life, though the healthy infant gut continues to undergo dramatic composition changes before stabilising at about age three or four [35]. Most studies agree that the infant gut microbiome closely resembles the adult gut microbiome by about age 3 years [36, 37]. Conversely, some studies argue that there are differences between the adult and child gut microbiome, such as increased abundances of Firmicutes and Actinobacteria observed in children compared to adults [38]. In addition, decreased Bacteroidetes (in pre-adolescent children) [38] and increased numbers of Bifidobacteria were seen in paediatric samples (ages 1–18) [38–40]. Potential differences in the microbiome of healthy children when compared to adults may also suggest that the microbiome of paediatric IBD may potentially differ to adult IBD microbiomes.

## The Gut Microbiome in Crohn’s Disease and Ulcerative Colitis

While the exact role the microbiome plays in IBD is unclear, many studies acknowledge the complex relationship between the gut bacteria and pathogenesis of IBD, as reviewed by Wilson and Russell [41]. Various studies have investigated the unique aspects of CD and UC in paediatric patients. Increased numbers of *Escherichia coli* were observed in paediatric CD stool samples but not in UC [42]. *Faecalibacterium prausnitzii* has been observed to be decreased in paediatric CD stool samples [42, 43] and increased in colonic biopsies [44] of paediatric CD compared to UC and controls. In contrast, Olbjørn and colleagues found only *Mycoplasma hominis* differed between paediatric CD and UC. Furthermore, the representatives of families Lachnospiraceae and Coriobacteriaceae were observed to be decreased in both paediatric CD and UC compared with controls, as well as the *Alistipes* species. Many studies have found differences between severe and milder cases of IBD, and others have seen differences between patients pre- and post-treatment. These findings have made it clear that there is a complicated and multifaceted relationship between the disease and the microbiome, though whether the microbiome plays a cause or an effect role, or perhaps a mixture of the two, has yet to be definitively proven.

### Changes in IBD from a Healthy Microbiome

As has also been observed in adult cohorts, children with IBD have a different microbiome than their healthy counterparts. A number of different bacteria have been found to be significantly changed in paediatric IBD patients, compared to controls. Table 1 contains a number of bacteria which have been found to be differentially abundant in paediatric IBD patients when compared with controls, as well as highlighting some of the microbial differences seen between UC and CD, and between varying disease states. Among those found to be decreased in paediatric IBD compared with controls are members of the *Bifidobacterium* genus [29, 42, 43, 45], which are commonly considered gut commensals and markers of a healthy gut. Specifically, both *Bifidobacterium longum* and *Bifidobacterium pseudocatenulatum* were decreased in paediatric IBD patients [45]. Most commonly reduced in paediatric IBD subjects are bacteria from the Firmicutes phylum, including the family *Ruminococcaceae*, and the genera *Eubacterium* and *Clostridium* [29, 42, 43, 45, 46], consistent with findings in adult IBD [47].

**Table 1:** Differences in bacterial abundance between A. paediatric IBD and controls, B. Paediatric IBD disease states, C. CD and UC

A. Bacterial abundance in paediatric IBD compared with controls			
*Ruminococcaceae* (*Faecalibacterium prausnitzii*, *Oscillospira*, and unclassified *Ruminococcaceae*)	Increased abundance in CD	Assa et al. [49]	Ileal Biopsies
*Enterobacteriaceae* (*Escherichia coli*), *Fusobacteriaceae* (*Fusobacterium nucleatum*), *Gemellaceae* (*Gemella moribillum*), *Neisseriaceae* (*Eikenella corrodens*), *Pasteurellacaeae* (*Haemophilus parainfluenzae*), *Veillonellaceae* (*Veillonella parvula*)	Increased abundance in CD	Gevers et al. [48]	Mucosal tissue biopsies (terminal ileum and rectum)
*Bacteroidales* (*Bacteroides vulgatus*, *Bacteroides caccae*), *Bifidobacteriaceae* (*Bifidobacterium bifidum*, *Bifidobacterium longum*, *Bifidobacterium adolescentis*, *Bifidobacterium dentum*), *Clostridiales* (*Blautia hansenii*, *Ruminococcus gnavus*, *Clostridium nexile*, *Faecalibacterium prausnitzii*, *Ruminoccus torques*, *Clostridium bolteae*, *Eubacterium rectale*, *Roseburia intestinalis*, *Coprococcus comes*), *Erysipelotrichales*	Decreased abundance in CD		
*Veillonellaceae*, *Streptococcus*	Increased abundance in CD		Faecal samples (small subset of cohort with stool samples) Faecal samples (small subset of cohort with stool samples)
*Clostridiales* (*Dorea*, *Blautia*, *Ruminococcus*)	Decreased abundance in CD		
*Enterococcus*	Increased abundance in CD	Kowalska-Duplaga et al. [43]	Faecal samples
*Bifidobacterium* (*Bifidobacterium adolescentis*), *Adlercreutzia*, *Clostridium* (*Clostridium celatum*), *Coprococcus*, *Roseburia* (*Roseburia faecis*), *Faecalibacterium* (*Faecalibacterium prausnitzii*), *Gemmiger* (*Gemmiger formicilis*), *Ruminococcus* (*Ruminococcus bromii*), *Veillonellaceae* (*Dialister*)	Decreased abundance in CD		
*Lachnospiraceae*, *Coriobacteriaceae*, *Bifidobacteriaceae* (in particular *Bifidobacterium longum* and *Bifidobacterium Pseudocatenulatum*)	Decreased abundance in IBD (UC and CD)	Maukonen et al. [45]	Faecal samples
*Bacteroides*	Increased abundance in UC		
*Lactobacillus*	Decreased abundance in UC		
Bacteroidetes, *Eubacterium*, *Prevotella*, *Alistipes* (*Alistipes finegoldii*, *Alistipes putredinis*)	Decreased abundance in IBD (UC and CD)	de Meij et al. [46]	Faecal samples
*Akkermansia muciniphila*	Decreased abundance in UC		
*Escherichia coli*	Increased abundance in IBD (UC and CD)		
*Prevotella*	Increased abundance in IBD (UC and CD) and symptomatic non-IBD	Olbjørn et al. [29]	Faecal samples
*Eubacterium rectale*, *Eubacterium biforme/Streptococcus agalactiae*, *Parabacteroides*, *Bifidobacterium*	Decreased abundance in IBD (UC and CD) compared with symptomatic non-IBD controls		
*Bifidobacteriaceae*	Decreased abundance in UC (active only)	Schwiertz et al. [42]	Faecal samples
*Faecalibacterium prausnitzii*, *Bifidobacteriaceae*	Decreased abundance in CD		
*Escherichia coli*	Increased abundance in CD (active only)		
B. Bacterial abundance differences between disease states in paediatric IBD			
*Ruminococcus gnavus*	Increased in IBD patients with extensive disease (ileocolitis in CD or extensive colitis in UC) compared to CD patients with isolated colonic disease and UC patients with left-sided colitis or proctitis	Olbjørn et al. [29]	Faecal samples
*Veillonella*	Increased in CD patients with upper gastrointestinal involvement compared to those without upper gastrointestinal lesions		
Proteobacteria	Increased abundance for CD patients with complicated disease behaviour, stricturing, or penetrating disease		
*Ruminococcaceae*, *Lachnospiraceae*, *Streptococcus anginosus* (only in rectal biopsies), *Veillonella dispar*, *Aggregatibacter segnis*, *Campylobacter*, *Lachnospiraceae*, *Veillonella parvula*, *Haemophilus parainfluenzae*, *Megasphaera*	Decreased in severe disease in UC	Schirmer et al. [30]	Faecal samples and paired rectal biopsies (study found comparable abundances between stool faecal samples and biopsies)
*Veillonella dispar*, *Veillonella parvula* (additional OTU depletions also found)	Decreased in UC with extensive disease involvement or pancolitis compared to UC with proctosigmoiditis or left-sided colitis		
*Clostridiales*, *Veillonella dispar*, *Haemophilus parainfluenzae*, *Campylobacter*	Increased in UC patients later requiring colectomy		
*Ruminococcaceae*, *Blautia*, *Dorea* (additional OTU depletions also found)	Decreased in UC patients later requiring colectomy		
C. Bacterial abundance in paediatric CD compared with UC			
*Akkermansia muciniphila*	Decreased abundance in UC	de Meij et al. [46]	Faecal samples
*Mycoplasma hominis*	Decreased in CD (compared to UC)	Olbjørn et al. [29]	Faecal samples

Although many studies do report similar findings in terms of which bacterial changes are seen, there are a small number of cases where findings differ across studies. For example, *Ruminococcaceae* is generally reported as reduced in IBD subjects [45, 48] but in Assa et al., an increase was seen in those with IBD [49]. Of course, inter-study variation may partially be a result of differences between the study designs, such as differences in DNA extraction methods, sequencing, analysis, and of course the type of sample, be that a stool sample or a biopsy (from varying locations) [50]. Moreover, a large inter-continental study of adult IBD recently highlighted geographical changes, in terms of different lifestyle, diet and ethnicity, to account for much of the observed microbiota heterogeneity [31].

Although a reduction in microorganisms considered ‘beneficial’ is the most striking difference of the microbiome in paediatric IBD, other species appear to colonise the paediatric IBD gut microbiome. *Escherichia coli* has often found to be increased in paediatric IBD vs controls [42, 46, 51]. Interestingly, a number of studies have found bacteria typical of the oral microbiome to be increased in paediatric IBD vs controls, and in cases of severe vs mild IBD in children [29, 30, 48]. There is speculation that these species may be taking advantage of the changed environment, or potentially exasperating or driving (by inducing T helper cells) inflammation in the guts of patients with IBD [30, 52, 53]. Many of these bacteria are anaerobic, such as *Veillonellaceae* and *Fusobacterium*, or facultative anaerobes, such as *Haemophilus parainfluenzae*, a member of the *Pasteurellaceae* family.

### Disease States and Severity in Paediatric IBD

Variation in microbial composition can also be seen across disease states and varying with disease severity, where the gut microbiome of children with mild IBD appears closer to healthy microbiome than for more severe IBD cases. Disease severity is often evaluating using specific scoring metrics such as the Paediatric Crohn’s Disease Activity Index (PCDAI) [54], the Mayo score [55], and the Paediatric Ulcerative Colitis Activity Index (PUCAI) [56]. These scoring systems generally take into account the impact of the disease on the patient, the disease burden, and disease course [57]. For instance, *Ruminococcus gnavus*, a gut coccus with a potential inflammatory role [58], was increased in children with total or ileocolitis compared to those with colonic CD or left-sided UC [29]. This taxa was also particularly enriched in the guts of paediatric IBD patients with increased disease activity [59]. Henke and co-authors proposed that this may be a result of a glucorhamnan polysaccharide synthesised and secreted by *Ruminococcus gnavus*, thereby promoting inflammation through the induced secretion of TNFalpha, which is dependent on the toll-like receptor 4 (TLR4) [58].

Furthermore, *Veillonella* was increased in paediatric CD patients who had upper gastro manifestations [29]. Specific strains of these, *Veillonella dispar* and *Veillonella parvula*, as well as other oral cavity bacteria such as *Haemophilus parainfluenzae*, were also found to be increased in severe disease [30]. Interestingly, *Haemophilus parainfluenzae* was detected at high levels in severe UC disease with drastic reductions over treatment time, and appeared to correlate with paediatric patients who would then enter remission. However, children with mild disease and less but consistent abundance of *Haemophilus parainfluenzae* were less likely to enter remission. Schirmer and colleagues hypothesised that this may mean a decrease in certain key bacteria, such as *Haemophilus parainfluenzae*, which may positively influence disease outcome.

## Potential Microbiome-Related Therapeutics, Diagnostics, and Prognostics

Although there is no cure available for CD or UC, a number of treatments are available for inducing remission. These treatment options vary depending on disease severity and disease type, and often have a significant effect on the microbiome. For mild to moderate IBD, 5-aminosalicylic acid (5-ASA) or antibiotic therapy have been used to induce remission [5], which in some studies appears to affect the gut microbiome [30]. The same authors also implicated several bacterial taxa, previously associated with disease severity, with antibiotic usage in paediatric UC patients [30]. These results were echoed in a previous study on paediatric CD that found antibiotic usage to increase microbiome dysbiosis in CD [48]. Antibiotic usage was also associated with both bacterial and fungal dysbiosis in a cohort of paediatric CD patients by Lewis et al. [60]. These findings, along with studies examining antibiotics as potential causative agents in the general paediatric population [61] and observational studies linking antibiotic usage in children to an increased risk of developing IBD [62, 63], highlights the need for further study of the long-term effects on paediatric IBD gut post-antibiotic-induced remission.

Corticosteroids, biologic therapy (anti-TNF agents such as infliximab, often for non-responders to traditional treatments), and thiopurines are used to treat children with UC and CD for moderate to severe disease [5]. Steroid treatment, however, comes with additional risk for paediatric patients, as it is imperative to avoid stunting growth in children who may already be at risk of growth retardation due to malnutrition, particularly patients with CD. Schirmer and colleagues also found 47 taxa associated with corticosteroids, whereof a majority were correlated with both remission and non-remission groups, raising the question of whether these changes in microbial composition are a by-product of treatment, or a driving factor in the efficacy of the treatment of IBD [30]. The interaction between biological therapies, such as anti-TNF, and the microbiome has not been extensively documented in paediatric IBD patients. However, Firmicutes and *Mycoplasma hominis* were decreased in paediatric IBD patients treated with biologic therapy compared with conventional methods (EEN in CD, and steroids and 5-ASA in CD and UC) [29]. No reduction in fungal colonisation in paediatric CD following treatment with anti-TNF has been observed [60].

In addition to medication-based treatments, both diet interventions and surgery are commonly used in the treatment of IBD. Special diets are found to help in CD, such as exclusive enteral nutrition (EEN), partial enteral nutrition (PEN) with a free diet, and CD exclusion diets (CDED). In Europe, EEN is currently recommended as first-line treatment for new cases [64]. These diets can have a profound effect on the microbiome, and microbial changes associated with treatment diets have been found in a multitude of IBD studies. Lewis and co-authors found that the microbiome composition of paediatric CD patients who responded to EEN was more similar to healthy controls than those who did not respond to EEN treatment. The authors also observed decreased fungal colonisation after EEN treatment [60] and *Haemophilus* species (associated with disease severity in paediatric UC [30]). The genus *Alistipes* was further increased in children receiving EEN but negatively associated with antibiotic treatment. In particular, *Alistipes finegoldii*i and *Alistipes putredinus* were decreased in paediatric CD patients, but these increased with EEN treatment (EEN was only given to CD patients) [46]. *Escherichia coli* has also been reported to be negatively associated with EEN treatment, indicating a shift towards a more ‘healthy’ microbiome with this treatment [46]. The authors also observed these trends in UC patients, who were treated with either aminosalicylates or aminosalicylates and corticosteroids. It is worth noting that those on EEN in this study were also receiving thiopurines [46]. In contrast, Quince and colleagues found that the microbiota did not return to that seen in healthy controls after successful treatment of CD with EEN, but to a distinct state [65].

Although EEN is an effective treatment for CD in paediatric patients (especially those with mild to moderate disease), one major shortcoming of the treatment is implementation due to low tolerance of the diet. Due to the challenges of maintaining a liquid diet for the time required, as well as a possible reversion seen when returning to a ‘free’ diet, a recently proposed treatment regimen is a so-called CD exclusion diet (CDED), which involves a whole food diet combined with partial enteral nutrition (PEN). Levine and colleagues reported that EEN and CDED resulted in similar levels of remission achievement at week 6 of a 12-week study, but CDED was more likely to have sustained remission at week 12 [66]. Similar species alterations were observed in the two treatment groups at week 6 (decreases in Proteobacteria, *Haemophilus*, *Veillonella* (which may be linked to lactate presence as these are lactate-utilising bacteria), *Bifidobacterium*, *Prevotella*, and *Anaerostipes* and increases in *Oscillibacter* and *Roseburia*). However, at week 12, those on EEN appeared to rebound to pre-treatment levels. These divergences once EEN patients began to transition back to a free diet suggest that CDED may be a more sustainable treatment strategy for paediatric CD [66]. Another study investigating CD exclusion diets in IBD had similar findings regarding achieving remission in EEN and CDED in adult CD subjects and a small number of paediatric CD subjects [67].

Surgery is often a treatment option of last resort for refractory disease. Twenty-one OTUs (operational taxonomic unit, represents a taxonomic unit of a bacterial species or genus, clustered by sequence similarity) were associated a colectomy being required in later treatment for paediatric UC patients, and three of the OTUs that were increased were oral bacteria, which were also associated with severe disease. Twelve of those decreased were also depleted in those with severe disease [30]. Higher abundances of Proteobacteria and lower *Faecalibacterium prausnitzii* were found to be associated with surgery and a lack of mucosal healing in paediatric IBD [29]. Decreased levels *Faecalibacterium prausnitzii* have also been observed in resected adult IBD patients [31]. The findings in these studies may suggest that certain bacteria could be used as identifying markers in patients who would become medical non-responders at a later point.

Faecal microbiota transplantation (FMT) may also be a potential treatment option for paediatric IBD, though it is currently not widely utilised [68]. Adult IBD studies (CD and UC) have shown success using FMT, with some patients achieving remission or showing a clinical response [69, 70]. Although a smaller number of studies are available for paediatric CD and UC, there has been some therapeutic potential suggested [71, 72]. However, other studies found that although safe, FMT did not provide any clinical benefit for some patients (adult mild to moderate UC [70], and paediatric UC [73]).

## The Virome

The viruses in the gut (the gut virome) have not been studied to the same degree as the bacteria in the gut, including those studies targeting IBD. However, it has been suggested that different abundances in the bacteriophages in the gut may also play a role in IBD [74]. The viral taxa found to be most abundant in the gut of both children and adults, irrespective of disease status, were *Caudovirales* and *Microviridae* [74, 75]. Increased richness and diversity of *Caudovirales* was observed in adult IBD compared with controls [74], and increased *Caudovirales* abundance has been reported in paediatric CD compared with UC [75]. This increase in *Caudovirales* was not detected in paediatric CD compared with healthy controls [60]. While increased richness of *Microviridae* in controls was observed compared to paediatric CD [75], this was not replicated in a more recent study, where no increased richness in adult UC or CD was seen when compared to controls [76]. The authors did however report increased diversity of *Caudovirales* in CD patients, but this was not seen in UC. Here, a significantly increased number of temperature phages in CD when compared with healthy controls [76].

## Mycobiome

The fungal microbiota, or mycobiome, though also less investigated than the bacterial microbiota, may show differences in the IBD gut when compared with healthy individuals. While several number of studies have observed changes in the fungal profile of IBD patients compared to healthy controls [60, 77, 78], these changes are not uniform between studies. An increased fungal abundance overall was observed in paediatric CD [60], and an increased fungal biodiversity was reported in CD, but not UC [77]. In contrast, Chehoud and colleagues reported reduced fungal diversity in paediatric IBD [79]. An adult cohort [77] and a small paediatric cohort [78] both reported Basidiomycota to be more abundant in IBD, with a corresponding decrease in Ascomycota. *Malassezia* (primarily *Malassezia restricta*) was found to be associated with CD, and responsible for the increase in basidiomycetes seen in the Limon et al. cohort of adult CD patients. *Malassezia restricta* was linked to adult CD patients with a disease-linked polymorphism in CARD9, and was found to exacerbate colitis in mouse models [80]. However, another study found fungi from the Ascomycota phylum to be increased in paediatric CD [60], as well as *Candida albicans*, which was also increased in adult IBD [77]. Another taxa, *Cyberlindnera jadinii*, was increased in paediatric IBD compared with controls [60, 79].

## Oral Microbiome

Though UC is generally limited to the colon, CD can occur anywhere in the gastrointestinal tract, and can include involvement of the oral cavity, with approximately 40% of a cohort of Irish paediatric CD patients (over a 3 year period) having oral involvement [81]. A significant decrease in diversity was seen in tongue samples of paediatric CD patients when compared to healthy and though non-significant, a decrease was also seen in the buccal samples of these paediatric CD patients [82]. Tongue samples in CD also showed a decrease in *Fusobacteria* and Firmicutes, but no phylum changes were observed in the buccal samples [82]. No difference in diversity was observed in UC patients in the tongue or buccal samples when compared with healthy, but certain bacterial changes were observed in tongue samples, namely a decrease in Fusobacteria and increased levels of Spirochaetes, Synergistetes, and Bacteroidetes [82]. As previously mentioned, increased numbers of oral bacteria have been observed in the gut of IBD vs healthy individuals, and in IBD patients with increased disease severity. This could suggest that an invasion of oral bacteria to the gut may contribute to part of the dysbiosis observed.

## Conclusion

Paediatric IBD is a lifelong condition (currently with no medical cure) and can have a severe impact on the quality of life of those suffering from the disease. The incidence and prevalence of paediatric IBD is rising, for both CD and UC, and the prevalence is particularly high in industrialised countries. The aetiology of IBD is unknown though it is thought that there is a genetic element, as well as environmental risks, such as industrialisation, and a western lifestyle. The microbiome is also believed to contribute to the risk of developing IBD.

Studies have also shown that both CD and UC have microbiomes that are significantly different from healthy individuals and it is clear from the literature that the microbiome and IBD have a varied and complex relationship. There is some disagreement on whether the healthy paediatric microbiome is identical to the adult microbiome, with arguments on both sides. However, both adult and paediatric IBD exhibit significant differences between disease and healthy controls. There is a decrease in bacterial diversity seen in the guts of IBD patients compared to controls. A number of bacteria, particularly commensals, have been reduced in paediatric IBD compared with controls, such as the *Bifidobacterium* genus and those in the Firmicutes phylum, such as those from the genera *Eubacterium*, *Ruminococcaceae*, and *Clostridium*. *Escherichia coli* and various oral bacteria, such as *Veillonellaceae*, *Fusobacterium*, and *Haemophilus parainfluenzae* appear to be increased in paediatric IBD.

Significant differences in microbial composition are evident in the different disease states and disease severity levels, like oral cavity bacteria (such as *Haemophilus parainfluenzae*) which were found to be increased with disease severity. Possible future research in this area may focus on whether the bacterial changes seen with increased disease severity are an outcome of the worsening disease or if the bacteria have the potential to be contributing to the changes in disease state or severity.

Treatments for IBD appear to have at least some impact on the microbiome, whether that is an intentional effect or a by-product of treatment. Antibiotics have been found to increase dysbiosis in both paediatric CD and UC, and microbes associated with disease severity have also been linked to antibiotic usage by studies. Both 5-ASAs and corticosteroids have been linked to changes in the microbiome and biological therapies such as anti-TNF may be linked to decreases in Firmicutes. Diet therapy, such as EEN, is extensively used as a treatment in paediatric CD. EEN was found to move paediatric CD patients closer to a healthy control microbiome in some studies, but to a distinct pattern of microbiota in others, and some bacteria were also reported to be changed with EEN therapy. An emerging diet therapy, CDED, was found to have similar microbiome changes as EEN treatment (such as Proteobacteria decreases). The CDED study shows promise for dietary treatments in paediatric patients without resorting to an entirely enteral diet.

Investigation of the oral microbiome is also worth considering as up to 40% of paediatric CD patients in one study had oral involvement. Decreases in diversity were found in tongue samples from paediatric CD, and both CD and UC had decreased *Fusobacteria* in tongue samples when compared to controls. Given the number of studies identifying oral bacteria in the gut of paediatric IBD patients, research into whether the oral bacteria may be invading the gut and contributing to the dysbiosis observed may be a future area of interest.

Less well studied, but a potential future area of interest are the other gut biomes in IBD: the virome and the mycobiome. The virome in IBD is a relatively unexplored area of research, and some disparity exists between the results so far, such as the possibility of increased *Caudovirales* in IBD. Though currently not extensively explored, a number of studies suggest that there may be significant changes in the mycobiome of IBD patients, and that fungal microbes may be more abundant in CD. Increased Basidiomycota has been reported in paediatric IBD patients, as well as increased abundances of species such as *Cyberlindnera jadinii* and *Candida albicans*.

Research into the paediatric IBD microbiome over the last number of years has yielded a large amount of new knowledge about the role microorganisms may play in the aetiology and pathology of CD and UC. These studies have great clinical significance and the accumulation of this knowledge is essential to gain a deeper understanding of the development and mechanisms of these serious conditions, as well as being crucial for the development of advancements in the treatment options for CD and UC.
